# A scoping review of instruments measuring the implementation of the Health-Promoting School approach

**DOI:** 10.1093/heapro/daag124

**Published:** 2026-08-13

**Authors:** Stefano Delbosq, Kathelijne Bessems, Marta Branda, Kevin Dadaczynski, Gerjanne Vennegoor, Karina Leksy, Anne Torhild Klomstén, Veronica Velasco

**Affiliations:** Department of Psychology, University of Milano-Bicocca, Milan, Italy; Department of Health Promotion, NUTRIM-Institute of Nutrition and Translational Research in Metabolism, Maastricht University, Maastricht, The Netherlands; Department of Psychology, University of Milano-Bicocca, Milan, Italy; Faculty of Human Science, Department of Sport and Health Sciences, University of Potsdam, Potsdam, Germany; Centre for Applied Health Sciences, Leuphana University of Lueneburg, Lueneburg, Germany; Department of Health Promotion, NUTRIM-Institute of Nutrition and Translational Research in Metabolism, Maastricht University, Maastricht, The Netherlands; Department of Social Science, Institute of Pedagogy, University of Silesia, Katowice, Poland; Department of Education and Lifelong Learning, Norwegian University of Science and Technology, Trondheim, Norway; Department of Psychology, University of Milano-Bicocca, Milan, Italy

**Keywords:** Health-Promoting Schools, evidence-based health promotion, scoping review, assessment tool, evaluation, implementation

## Abstract

The Health-Promoting School (HPS) is a holistic approach that addresses not only the curriculum but also policies, the physical and social school environment, and the school’s external environment. Given its complexity in implementation, evidence on effectiveness is mixed. Adequately measuring the implementation of the approach is therefore essential for research and practice. This paper presents the results of a scoping review of instruments that measure the implementation of the HPS approach at the school level. The review was conducted using databases (Scopus, Web of Science, and EBSCO Global Health) and a survey targeting experts and stakeholders in school health promotion to collect unpublished instruments. The instruments were analysed with respect to the HPS components and health topics addressed, and implementation indicators. In total, 28 instruments were retrieved. Heterogeneity in instrument length, recipients, data collection methods, theoretical orientations, and methodological quality in the development and validation was observed. In relation to the HPS model, ‘Healthy school policies’ was the most frequent component, while ‘School Social Environment’ was the least addressed. Implementation indicators (implementation outcomes and steps in the implementation process) were present but were mostly not grounded in theoretical frameworks. Among the health topics, ‘nutrition’ and ‘physical activity’ were most frequently addressed for students, while ‘relationships’ was most often found for school staff. For the first time, the study provides an overview of existing instruments to capture the implementation of different components/indicators of HPS. Its results offer room for consolidation and further development of this emerging field.

Contribution to Health Promotion

- This review streamlines existing efforts to capture the implementation of different components/implementation indicators of Health-Promoting Schools (HPSs) for the first time.
- This review offers room for discussion about consolidation and further development of implementation instruments and items of this emerging field, for instance, by further distinguishing HPS components.
- Future research should develop instruments adopting methodologically sound processes to develop and validate the instruments.
- Instruments should include more items related to the implementation processes (e.g. cyclical planning and evaluation) and outcomes (e.g. dose, adherence, and participant responsiveness) of the HPS approach.
- Instruments should also better address the teaching and non-teaching school staff.

## Introduction

A Health-Promoting School (HPS) is described as a school that is constantly strengthening its capacity as a healthy setting for living, learning, and working (WHO and UNESCO 2021a). As Gugglberger (2021) noted, the adoption of the HPS approach could be related to a wide range of health promotion (HP) topics. This approach is in line with the Ottawa Charter, which suggests integrated setting-based approaches, including the school setting, by intervening on building public health policy, creating supporting environments, strengthening community action, fostering the development of personal skills, and reorienting services as strategic HP action areas (WHO 1986).

Despite its worldwide recognition (Turunen *et al*. 2017, Jullien *et al*. 2025, WHO 2025), there is mixed evidence on the effectiveness of the HPS approach. Most studies consider changes in health outcomes (e.g. behaviours or states of well-being) or social outcomes (Nutbeam 1996) as endpoints of the HPS approach. The HPS approach has been shown to be effective for certain, but not all, student health outcomes and behaviours, although with small effects and inconsistently across studies (Langford *et al*. 2015b, Abuhaloob and Petersen 2023). Furthermore, there are studies showing no difference in health outcomes between students from HPS and non-HPS schools (McIsaac *et al*. 2017, Hayek *et al*. 2019) or even opposite effects (Sánchez-Hernando *et al*. 2022).

This heterogeneity can be partially explained by the complexity of the HPS approach and its implementation (In this paper, the term ‘implementation’ is defined as the conduct of a specified set of activities to establish or put in place a programme or initiative (Fixsen *et al*. 2005).) (Langford *et al*. 2015b, 2017), including different factors (discussed below):

1. the conceptualizations of the HPS approach and its implementation (theoretical frameworks encompassing different components and the adopted guidelines and manuals used to guide implementation);
2. the contextual dimensions related to the educational systems, the communities, and the schools; and
3. implementation by schools.

First, the development of the HPS approach varied across and within countries, with different names and conceptualizations, such as whole-school approach, Comprehensive School Health, Child Friendly Schools, and the FRESH initiative (IUHPE 2008). This has led to a variety of components to indicate what the approach includes and how it should be addressed by policymakers, schools, and researchers. For instance, a conceptualization with three components (school curriculum, ethos and environment, and families and communities) was used in the Cochrane systematic review by Langford and colleagues and in other studies (Langford *et al*. 2014, Lekamge *et al*. 2025b). Another framework, Whole School, Whole Community, Whole Child (WSCC; ASCD and CDC 2014), lists 10 components. However, six key HPS components have been widely recognized and used by relevant international stakeholders (IUHPE 2008, Vilaça *et al*. 2019, WHO and UNESCO 2021a). This conceptualization includes healthy school policies, physical environment, social environment, individual health skills and action competencies, community links, and health services (IUHPE 2008, Vilaça *et al*. 2019, WHO and UNESCO 2021a).

Second, HPS implementation is challenging because schools are complex adaptive systems that all react uniquely to HP activities and in which achieving successful change thus depends on many intertwined factors and causal paths (Keshavarz *et al*. 2010, Rosas 2017). Contextual dimensions, such as the organizational and structural changes necessary for implementation, and the characteristics of the school and its members (e.g. from children to school leaders) have an influence on the implementation and hence its effectiveness (Langford *et al*. 2015a, Darlington *et al*. 2018, 2020, Bartelink *et al*. 2019, Lee *et al*. 2020, Vennegoor *et al*. 2025). Furthermore, schools implement the HPS approach according to their context-specific values, needs, competences, and resources (Bartelink and Bessems 2019, van Dongen *et al*. 2023), thus making monitoring of HP activities (In this paper, the term ‘HP activity’ is used for the activities, strategies, policies, and programmes that a school adopts to promote health, including prevention strategies (WHO and UNESCO 2021a).) more complex.

Finally, the literature has stressed the importance of investigating and considering the quality of HPS implementation at the school level (Samdal and Rowling 2011, Darlington *et al*. 2020, SHE Network 2020). This implies investigating different levels of HP actions and outcomes, such as the ones identified by Nutbeam (1996, 1998). Furthermore, this implies investigating implementation indicators such as implementation outcomes and processes (Fixsen *et al*. 2005, Proctor *et al*. 2011, Schaap *et al*. 2018). Implementation outcomes can be defined as ‘the effects of deliberate and purposive actions to implement new treatments, practices, and services’ (Proctor *et al*. 2011, p. 65). They include acceptability, adoption, appropriateness, feasibility, fidelity, implementation cost, penetration, and sustainability (Proctor *et al*. 2011). It is important to investigate implementation through each stage of the implementation process, from initially adopting the approach (Inchley *et al*. 2007, Vilaça *et al*. 2019) to sustained and cyclical HPS implementation (i.e. steps in the implementation of HP activities, phases such as needs assessment, planning, implementing, monitoring, and assessing the HP activities).

Considering these reflections, appropriate implementation indicators and methods are crucial for effective and sustained school HP (Joyce *et al*. 2017). With respect to the literature, in some studies, the implementation of the HPS components has been investigated through qualitative or mixed studies (Moynihan *et al*. 2016, Heesch *et al*. 2020). In others, it has been investigated quantitatively (Liao *et al*. 2015, Lee *et al*. 2019, Vennegoor *et al*. 2023b, Bartelink *et al*. 2024). Quantitative methods are particularly useful to evaluate HPS implementation on a larger scale, in an efficient and/or consistent way, or as a first step towards a deeper (qualitative) understanding of the implementation process. Quantitative instruments investigating the implementation of the HPS approach have assessed it in different ways. For instance, the School Rapid Assessment tool (Safarjan *et al*. 2013) is a checklist distinguishing different HPS components, while the instrument by Dadaczynski and Hering (2021) is a scale with the factors.

While we are aware of some instruments measuring the implementation of the HPS approach, a literature review was deemed necessary to expand knowledge in this research area. In fact, an overview of the operationalization of these instruments at the international level could provide insights into different kinds of implementation of the HPS model and foster research within the school setting (e.g. selecting appropriate indicators, developing monitoring processes, or administration modalities).

### Aim of the review

The aim of this review is to provide an overview of the existing instruments measuring the implementation of the HPS approach at the school level.

The research questions are

RQ1) Which instruments measure the implementation of the HPS approach at the school level and what are their characteristics (e.g. country of origin, developers, subdivisions, response scale, recipients)?

RQ2) How is the HPS approach operationalized within the instruments?

•RQ2.1. Which HPS components do they cover?
•RQ2.2. Which implementation indicators (i.e. implementation outcomes and steps in the implementation process) do they cover?

RQ3) Which health topics are covered by the instruments?

## Materials and methods

### Approach

Since there is no overview of available instruments measuring the implementation of HPS, we conducted a scoping review to explore the international landscape (Munn *et al*. 2018), collecting data in two ways: by a database search and by a survey targeting experts and stakeholders in school HP (Arksey and O’Malley 2005). The survey was included because, although many countries monitor the implementation of the HPS approach quantitatively, findings are not necessarily published in the scientific literature.

### Information sources and search strategy

The literature search involved Scopus, Web of Science, and the EBSCO Global Health databases. Searches were conducted on 20 March 2024 on the first two databases, and on 16 May 2024 on the latter. The detailed search string is provided in Supplementary File 1. Since some instruments were cited in a document without providing detailed information about them, a backward citation strategy (Hirt *et al*. 2024) was adopted in order to retrieve instruments from the papers, documents, or reports in which they were actually reported. The search results were uploaded to the app Rayyan. Duplicates were removed by manually reviewing the records that the app function identified as potential duplicates. Sources from the survey were relatively few and were not uploaded to Rayyan. Supplementary Figure S1 illustrates the PRISMA flowchart of the scoping review.

The expert survey was an *ad hoc* questionnaire developed by the authors. The survey aimed to collect information about the implementation of the HPS approach in different countries and to collect instruments used to measure it. Recipients were national and regional coordinators and HPS researchers from the former School for Health in Europe (SHE) Network (The SHE network foundation and a research group with people with expertise with the aim to further developing, exploring, and testing relevant issues and approaches concerning school HP, involving around 150 potential participants from 51 countries/regions, including non-European stakeholders. The survey was administered through Qualtrics from October 2024 to April 2025. Former SHE coordinators and researchers were invited to participate through networks, and participation was extended to other school HP experts via a newsletter by the UNESCO Chair ‘Global Health & Education’ in November 2024. Information about the survey was also promoted more informally during an international HP conference. A reminder to participate in the survey was sent via email and researchers also personally contacted some of the possible participants. The Committee for Research Evaluation of the University of Milano-Bicocca approved the survey (approval: protocol no. RM-2024-832). Documents and links leading to documents reported in the survey were then evaluated during the selection process. As per the PRISMA flowchart, 21 responses were collected from 19 countries/regions (response rate for countries/regions: 37%).

### Selection process

The screening process for the identified documents consisted of two stages: the first stage involved a screening of the title and abstract, and the second stage consisted of a full-text screening by two reviewers. In case of doubt, agreement was reached by group discussion. Inclusion criteria were (i) reporting quantitative instruments (e.g. surveys and checklists); (ii) measuring the implementation of HP activities; and (iii) measuring activities at the school level. Exclusion criteria were (i)reporting qualitative studies (e.g. qualitative analysis from interviews, focus groups, or documents analyses); (ii) missing reference to a whole-school approach (i.e. exclusive focus on one component, e.g. only curricular activities); (iii) reporting instruments measuring only one or two specific health topic focus (such as nutrition or physical activity), since those would not cover the adoption of the HPS approach overall but only with respect to a single HP target. Therefore, instruments covering only the topic of nutrition or any other topic within the HPS approach were not included; (iv) reporting data not collected at the school level.

If more editions of an instrument were identified, the most recent one available was selected.

### Data extraction and analysis

Instruments in foreign languages obtained during the expert survey were translated into English by using online translators. In case of uncertainty, the translated version was sent to the developers/corresponding authors for validation.

Information collected included: name of the instrument, country and institution, recipients (e.g. school principals and teachers), structure and length (i.e. number of subdivisions and items), references to whole-school approach, developmental background, and validation (e.g. use of guidelines, consensus process, pre-testing, and factorial analysis).

Most instruments were divided into subdivisions. These divisions were described as specific scales or scale’s dimensions or were reported as separated blocks with specific headings. Some authors were contacted to collect information to clarify divisions within instruments.

Consequently, two levels of classification were applied: subdivision level to determine the coverage of HPS components and implementation indicators, and item level to determine the coverage of health topics.

Two researchers (S.D. and M.B.) independently examined the subdivisions and items within the instruments. Then, the two analyses were compared and discussed. The analytical procedure and results were shared, discussed, and updated within the research team on different occasions: after the collection of the literature, in *ad hoc* small meetings, and within the whole group after the inclusion of instruments from the expert survey.

No quality appraisal of the instruments was performed because of differences within instruments (e.g. some of the typical validity measures do not apply to checklists), lack of information (especially with respect to instruments from the expert survey), and consistency with the aim of the scoping review (Peters *et al*. 2020, Khalil and Tricco 2022). Content validity of the HPS approach was examined through the HPS component analysis.

#### Identifying the Health-Promoting School components and implementation indicators

An analysis identifying the HPS components covered by the instruments was conducted. The unit of classification was the subdivision. The subdivisions were classified into HPS components based on the content of the items that they were comprised of. If a subdivision relevantly covered more than one HPS component, it was classified under each of those components. Consideration was given to the relevance and proportion of the individual HP activities within subdivisions, with no fixed cut-off values being set for weighting them within a subdivision.

Activities were included if, in line with Nutbeam’s outcome model (1996, 1998), they were related to HP actions (e.g. activities related to education), direct HP outcomes (e.g. organizational practices), or intermediate health outcomes (e.g. healthy environments). This model was chosen since it is also used in other HPS frameworks (Lee *et al*. 2005). In contrast, we did not consider items addressing general information about the school (e.g. school type) or measuring health and social outcomes (e.g. health-related quality of life).

While there are different interpretations and classifications of the HPS components, we adopted the one provided by the SHE Network (Vilaça *et al*. 2019), in line with the expertise and the use of the model within the national and international contexts of the authors.

We adopted the widespread SHE Network model, which includes the following six components:

1. ‘Healthy school policies’ are accepted practices or clearly defined documents designed to promote health and well-being. The policies are part of the school plan.
2. ‘School physical environment’ includes changes in the buildings, grounds, and school surroundings.
3. ‘School social environment’ relates to the quality of the relationships among and between school community members, e.g. among students, and between students and school staff.
4. ‘Individual health skills and action competencies’ are promoted through the curriculum, such as school health education and activities that develop knowledge and skills which enable students to build competencies and take action related to health, well-being, and educational attainment.
5. ‘Community links’ include practices that constitute links between the school and the students’ families and the school and key groups/individuals in the surrounding community.
6. ‘Health services’ are the local and regional school health services or school-linked services that are responsible for the students’ health care and HP by providing them direct services.

As for the implementation indicators, we considered activities related to implementation outcomes (Dusenbury *et al*. 2003, Proctor *et al*. 2011, Schaap *et al*. 2018) and steps in the implementation process (Vilaça *et al*. 2019, WHO and UNESCO 2021b), such as nominating a responsible person for HPS activities or setting up working groups.

HP activities were considered with respect to the HPS component to which they pertained. For instance, ‘keeping the campus clean’ requires adopting specific routines, procedures, and practices. Therefore, it is considered under ‘Healthy school policies’ and not under ‘School physical environment’. Having a gym or having proper facilities for disabled students would constitute an example of an HP activity under ‘School physical environment’. While in many cases an HP activity operates in multiple components, we opted to select the one that was most directly related to the item’s content. This strategy was chosen to stay as close as possible to the phrasing of the HP activities, without projecting intentions or goals that may not have been intended by the original authors. In case of doubt, agreement was reached by discussing in the group.

#### Identifying health topics

An analysis was conducted to identify the health topics covered by the instruments’ items. Items were primarily classified according to a list of health topics and secondarily according to the six HPS components. For instance, ‘Balanced diet menu prepared’ relates to ‘Nutrition’ as a topic and ‘Healthy school policies’ as the HPS component. Instruments were described by the number of health topics they covered.

The topic list included: nutrition; physical activity; smoking, alcohol, and drugs; climate and environmental sustainability; prevention of communicable diseases—hygiene; health check-up; sexuality; social relations and bullying; mental health; first aid—medical emergencies; traffic safety; media literacy and use; and general HP for school staff.

## Results

### Characteristics of the instruments

Twenty-eight instruments were retrieved and analysed, 16 through the literature review and 12 through the expert survey. Table 1 reports the characteristics of each instrument in detail.

**Table 1:** Characteristics of the HPS implementation instruments.

#	Name	Collected from	Country	Created by/used by	Retrieved from	Administration modality	Recipient	Subdivisions	Items	References to whole-school approaches	Development and validation
1	Health-Promotion Scale	Literature review	Australia	McBride (2000)		Survey through interviews	School principal and/or health coordinator	1	9	Sources mentioning HPS	Scale reliability (Cronbach’s *α*: 0.79)
2	School organizational scale	Literature review						1	5	Sources mentioning HPS	Scale reliability (Cronbach’s *α*: 0.76)
3	School health index (middle and high school version)	Literature review	USA	Centers for Disease Control and Prevention (2017)		Survey	School health team	11	174	Based on WSCC	Developed by the CDC
4	HPS criteria for Bronze-level accreditation	Literature review	China	Tian (2005)	Xin-Wei *et al*. (2008)	Checklist	Self-assessment + team of education/health officers	6	69	Based on HPS	Adaptation of criteria by World Health Organization - Regional Office for the Western Pacific (WHO/WPRO) (1996)
5	Checklist HPS in Lao	Literature review	Lao	Yoshimura *et al*. (2009)		Checklist	Interviews (principals, pupils, vendor, community chief) and observations	5	64	Based on HPS	Adaptation of criteria by World Health Organization - Regional Office for the Western Pacific (WHO/WPRO) (1996) Pre-testing, scale reliability (Cronbach’s *α*: 0.75, 0.78, 0.79, 0.69, 0.58)
6	Checklist (HPS accreditation system in Northern India)	Literature review	India	Thakur *et al*. (2014)		Checklist (1–3 points, not specified)	Not clear	8	24	Based on HPS	Consensus process (literature review, stakeholder involvement, manual drafting, pilot testing, reliability analysis: 0.7)
7	Scale for HPSs (SHPS)	Literature review	South Korea	Lee *et al*. (2014)		Survey	School principals, teachers and counsellors	7	37	Based on HPS	Consensus process (based on guidelines, involvement of expert, pre-testing, exploratory factor analysis, confirmatory factor analysis, scale reliability with Cronbach’s *α*: 0.97, 0.86, 0.91)
8	Instrument from the Taiwan Health-Promoting School Accreditation System	Literature review	Taiwan	Chen and Lee (2016)	https://www.hpsinc.tw/page/Accreditation	Accreditation system (document review and on-site visit)	Operators	6	73	Based on HPS	Delphi process (use of documents and guidelines, involvement of stakeholders)
9	HPS Monitoring Questionnaire	Literature review	South Africa	Struthers *et al*. (2017)		Survey	Students	6	138	Based on HPS	Delphi process (based on other instruments and action areas by the Ottawa Charter, involvement of stakeholders, pre-testing, test–retest reliability with Cohen’s *κ*: mixed results)
10	Survey of School Promotion of Emotional and Social Health	Literature review	Australia	Dix *et al*. (2019)		Survey	Principals or delegates	4	13	Based on HPS	Delphi process (based on other instruments, involvement of stakeholders, exploratory factor analysis, confirmatory factor analysis, scale reliability with Cronbach’s *α*: 0.85)
11	HPS performance indicators (Hong Kong Healthy School Awards Scheme)	Literature review	Hong Kong	Lee *et al*. (2019)		Checklist (on-sit visit as described in Chen and Lee 2016)	Accredited operators	6	92	Based on HPS	Consensus process (use of guidelines, piloting, involvement of stakeholders and experts)
12	School health checklist (Kenyan Comprehensive School Health Program)	Literature review	Kenya	Akiyama *et al*. (2020)		Checklist + survey	Principals, teachers, and students	8	63	Based on HPS	Based on a revised version of items from Government of Kenya (2009)
13	HPS implementation instrument	Literature review	Germany	Dadaczynski and Hering (2021)		Survey	Principals	2	10	Based on HPS	Validity assessed with principal component analysis, scale reliability with Cronbach’s *α* (0.83, 0.87)
14	HPS Implementation Questionnaire	Literature review	The Netherlands	Vennegoor *et al*. (2023a)		Survey + checklist	School employee knowledgeable about the school’s approach to student health and well-being	7	28	Based on HPS	Delphi method (interviews, involvement of experts, pre-testing, confirmatory factor analysis, scale reliability with Cronbach’s *α*: 0.74, 0.89, 0.84, 0.74, 0.73, 0.90)
15	FRESH Monitoring and Evaluation (M&E) Guidance (School-level checklists)	Literature review	International	International: FRESH M&E Coordinating Group: monitoring and evaluation guidance for school health programmes. Focus Resources on Effective School Health (FRESH 2014). Paris: United Nations Educational, Social and Cultural Organization	Website of Save the Children: https://resourcecentre.savethechildren.net/document/monitoring-and-evaluation-guidance-school-health-programs-eight-core-indicators-support	Survey + checklist (observations, interviews, focus groups)	Operators, school principals, teachers, and students	4	54	Based on the FRESH framework	Developed by the FRESH M&E Coordinating Group. Piloting
16	HPS Implementation Status Questionnaire	Literature review	Taiwan	Chang *et al*. (2014)		Survey	Person in charge of the HPS programme	6	20	Based on HPS	Consensus process (based on guidelines, involvement of experts, pre-testing)
17	Indicators to evaluate Programa de Melhoria do Acesso e da Qualidade da Atenção Básica (from Brazil)	Expert survey	Brazil	Wachs *et al*. (2022)		External evaluation process with a checklist	Primary care teams (field workers, including coordinators and supervisors)	3 (divided based on the tables reported on the paper)	39	Generic school health promotion	Developed within the Programa Saúde na Escola do Programa de Melhoria do Acesso e da Qualidade da Atenção Básica
18	Questionnaire for the evaluation of the annual activities of health-promoting educational institutions	Expert survey	Latvia	Latvia’s Center for Disease Prevention and Control	https://www.spkc.gov.lv/lv/media/18357/download?attachment	Survey	Schools (with a designated education institution coordinator as contact person)	1	10	Based on HPS (questionnaire for an HPS network)	Development within the Latvian HPS network
19	Health Behaviour in School-aged Children (HBSC) questionnaire about school—Health Promotion 2022 Slovakia	Expert survey	Slovakia	Working group for the school-level questionnaire for the HBSC study	Sent by the authors	Survey + checklist	School leadership	5	24	Based on HPS	Developed within an institutional working group and stakeholder consultations for the school-level questionnaire within the Slovak HBSC study
20	Lista de comprobación y revisión de los estándares de las Escuelas Promotoras de la Salud	Expert survey	Spain	Escuelas Promotoras de Salud	Ministerio de Sanidad and Ministerio de Educación Formación Profesional y Deportes (2023)	Checklist	School leadership	6	64	Based on HPS	Developed within an institutional working group (Grupo de Trabajo de Escuelas Promotoras de Salud)
21	Holistic Health Promotion Questionnaire from Hungary	Expert survey	Hungary	Egészséges Magyarország 2014–2020	Website no longer available	Survey	School principal	5	118	Based on HPS	Developed by the Hungarian State Secretariat for Public Education and the State Secretariat for Health (Köznevelésért Felelős Államtitkárság és az Egészségügyért Felelős Államtitkárság)
22	SHRN School Environment Questionnaire	Expert survey	UK, Wales	The School Health Research Network (SHRN)	https://www.shrn.org.uk/national-data/	Checklist	School senior leadership team or school-appointed representative	10	115	Based on HPS	Developed by the SHRN
23	Global School Health Policies and Practices Survey	Expert survey	International	WHO and United Nations Educational, Scientific and Cultural Organization (World Health Organization & UNESCO, 2023)	https://www.who.int/teams/noncommunicable-diseases/surveillance/systems-tools/global-school-health-policies-and-practices-survey	Checklist	Primary and secondary school principals and head teachers	8	356	Based on HPS	Developed by the WHO
24	SHE Rapid Assessment Tool	Expert survey	International	School for Health in Europe Network Foundation	Safarjan *et al*. (2013)	Checklist	School leadership	7	37	Based on HPS	Based on the Healthy Eating and Physical Activity in Schools (HEPS) Rapid Assessment tool (Simovska *et al*. 2010)
25	HPS Lombardy Monitoring Instrument	Expert survey	Italy—Lombardy	University of Milano-Bicocca/Lombardy’s Health Promoting Schools Networks	Sent by the authors, a report on the measured health promotion practices (Velasco and Delbosq 2025)	Survey + checklist	School principal and/or health coordinator/HPS contact person	9	184	Based on HPS	HP activities selected via a consensus project for guidelines (Cabina di Regia regionale e Cabine di Regia Provinciali SPS Lombardia 2024), adapted for the instrument. Undergoing evaluation
26	Instrument from the Plataforma de candidatura ao Selo Escola Saudável	Expert survey	Portugal	Direção-Geral da Educação (2025) https://www.dge.mec.pt/noticias/candidatura-selo-escola-saudavel-2024-2025		Checklist	Coordinator teacher of Health Promotion Education (PES)/Projeto de Educação para a Saúde e Educação Sexual (PESES)	10	103	Within the PESES	Not Available (NA)
27	Collection of instruments from Gezonde School	Expert survey	The Netherlands	https://www.gezondeschool.nl/vignet-en-themacertificaten-gezonde-school; https://mijngezondeschool.nl/certificaten		Checklist and collection of documents	School leadership	6	615	Based on HPS (healthy school)	Developed by Gezonde School. For the purpose of the review different instruments to award certificates to different topics were considered together, as they were developed within a whole-school approach
28	Instrument for awarding the Labélisation EDUSANTE (Réunion version)	Expert survey	France	https://www.ac-reunion.fr/media/22210/download		Checklist and collection of documents	School leadership	4	92	Based on HPS	Developed within the initiative Ecole Promotrice de Santé, awarding the Labélisation EDUSANTE

**Figure 1:**
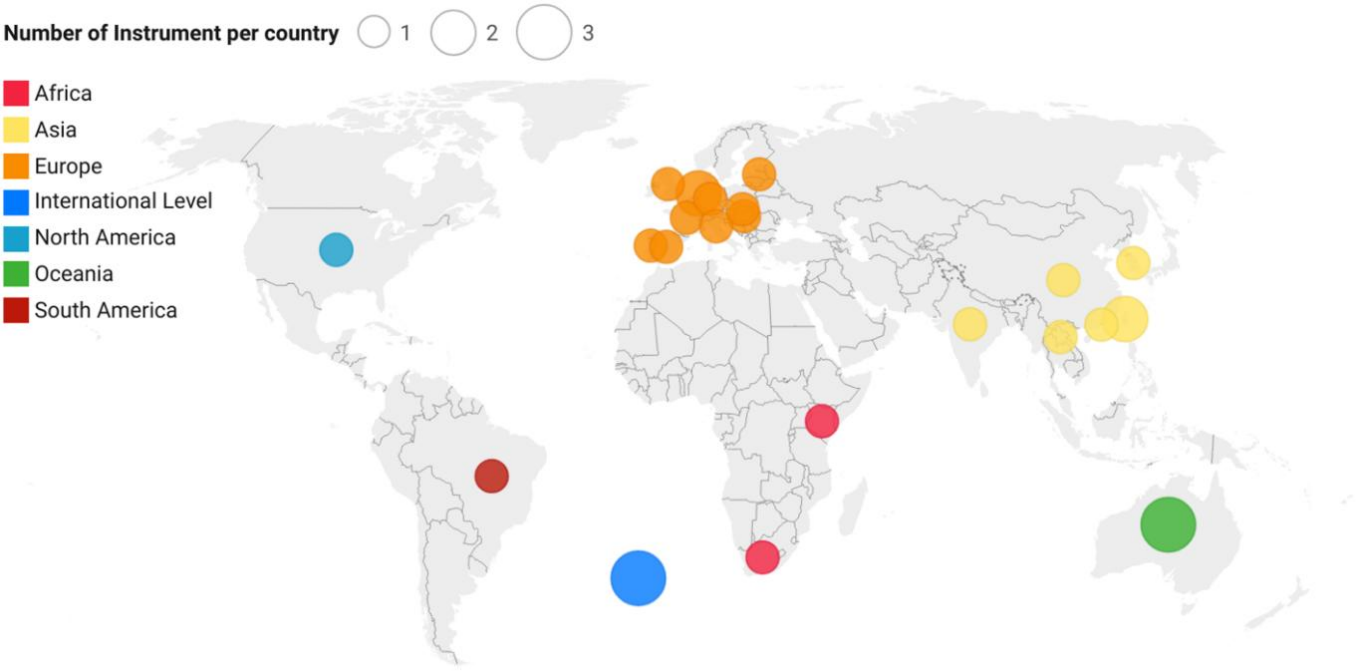
Number of instruments per country. Source: Compiled by the authors. Figure created using Datawrapper.

European and Asian countries were the most represented (11 and 7 instruments, respectively; see Fig. 1 and Table 1). Three instruments were developed by international entities: FRESH M&E Coordinating Group, WHO & UNESCO, and the SHE Network. Twenty-four instruments (86%) were developed within institutional entities (e.g. WHO and UNESCO) or intersectoral partnerships (e.g. established national or regional HPS networks involving schools, policymakers, and researchers), but only 6 (21%) employed complex evaluation systems involving multiple stakeholders and diverse data collection methods. While not many differences across continents were found, some instruments from low- and middle-income countries included HP activities related to health security and hygiene that were not included in instruments from high-income countries (e.g. water quality, Lao; broken windows in the classroom, South Africa; or preventing animals from having access to school, Kenya).

**Figure 2:**
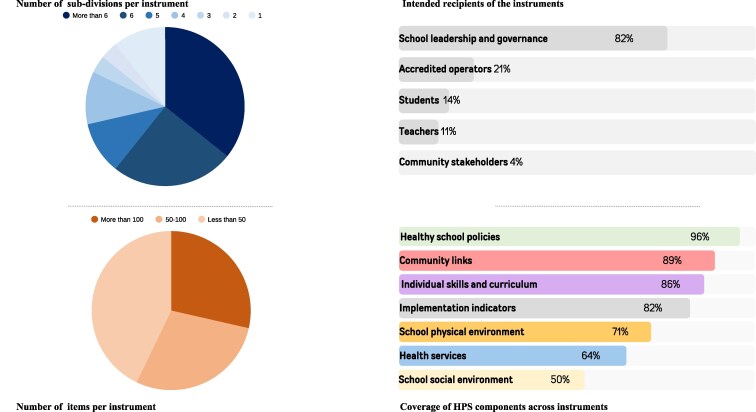
Overview on the characteristics of the instruments.

Most instruments (89%) were divided and contained between 2 and 11 subdivisions (*M* = 5.8, Fig. 2). The number of items ranged from 5 to 615 (*M* = 93.9), with most instruments comprising over 50 items (see Fig. 2). However, the instrument with the highest number of items was a collection of various questionnaires used for awarding certificates in different subject areas (see Table 1, instrument #27). Without it, the number of items ranged from 5 to 356, with a mean of 74.6.

Most instruments were administered through a survey (36%; Likert-scale responses), a checklist (46%; Yes/No responses), or both (18%). In some cases, such as accreditation systems, complex evaluation methods were used (data collection involving different stakeholders and mixed methods, such as interviews, observations, or surveys). The school leadership team (e.g. school principal, health coordinator, and HP working group/team) was the main recipient of the instruments, while teachers, students, or community stakeholders were less frequently addressed (see Fig. 2).

With respect to methodological quality, 12 instruments (43%) stated they had used guidelines or other instruments as sources for development. Eight instruments (29%) report some sort of pre-testing or piloting. Nine instruments (32%) reported reliability measures (such as Cronbach’s *α* or Cohen’s *κ*), while 10 instruments (36%) referred to some sort of consensus process (such as a Delphi approach) and 4 instruments (14%) examined the factorial structure (e.g. exploratory factor analysis or confirmatory factor analysis). Supplementary File 6 provides an overview of these characteristics by instrument.

### Health-Promoting School components

Overall, all HPS components were addressed, albeit with varying frequency (see Fig. 2). ‘Healthy school policies’ was the most present component, with all but one instrument covering it, while ‘School social environment’, was only found in half of the instruments. Ten instruments (36%, instruments #3–5, 7–9, 11, 21, 24, 25) included all the considered HPS components, and 7 of them also included practices related to implementation indicators. Of those 10 instruments, 7 were retrieved from the literature review and 3 from the expert survey. Heterogeneity emerged with respect to the number of subdivisions covering the components (i.e. in some instruments a component was covered in only one subdivision and in others, it was covered by many, see Supplementary File 2).

The 28 analysed instruments include a total of 162 subdivisions (see Supplementary File 2); 41 subdivisions (25%) were classified under only one HPS component/implementation indicators and 8 subdivisions (5%) were not classified (mostly because of items about descriptive information about the school or health and social outcomes).

#### Healthy school policies

Twenty-seven instruments included at least one subdivision covering ‘Healthy school policies’, and overall, 102 subdivisions were classified under this component, making it the most frequently represented component. Fifteen percent of the subdivisions classified in this component had names explicitly referring to school policies or planning in general (e.g. ‘Health and well-being policies at school’ and ‘Planning oriented toward health’) or together with school environment (e.g. ‘School health and safety policies and environment’ or ‘Structural and organizational environment’).

Twenty-eight subdivisions (27%) which included this component explicitly referred to health topics, such as nutrition, physical activity, mental health, prevention of diseases, smoking, alcohol and drugs, and social relations and sexuality.

In a considerable number of occurrences (*n* = 49, 48%), subdivisions intended to cover other components were also classified in ‘Healthy school policies’ (e.g. ‘Educational community and partners’, ‘Health education’, or ‘School’s social environment’).

#### Community links

Twenty-five instruments included at least 1 subdivision covering ‘Community links’, and overall, 48 subdivisions were classified under this component. One-third of the subdivisions classified in this component tended to be general (e.g. ‘School–community relationship’ or ‘Community partnerships’) were specifically related to families (e.g. ‘Family Engagement’) or were combined with ‘Health services’ (‘Collaboration with the community and services’).

Twelve subdivisions (25%) addressing community links referred to health topics, such as nutrition, physical activity, mental health, smoking, alcohol and drugs, and social relations and sexuality.

In some occurrences (*n* = 8, 17%), subdivisions intended to cover other components were also classified in ‘Community links’ (e.g. ‘Social and emotional climate’, ‘Health and well-being education and curriculum reform’, or ‘School physical environment’).

#### Individual skills and curriculum

Twenty-four instruments included at least 1 subdivision covering ‘Individual skills and curriculum’, and overall, 55 subdivisions were classified under this component. Half of the subdivisions classified in this component explicitly referred to health skills and education in general (e.g. ‘Health education’ or ‘Personal health and life skills’), or physical education specifically (e.g. ‘Physical education and physical activity programme’).

Eighteen subdivisions (33%) from this component addressed health topics, such as nutrition, physical activity, prevention of diseases, smoking, alcohol and drugs, and social relations and sexuality.

In some occurrences (*n* = 11, 20%), subdivisions intended to cover other components were also classified in ‘Individual skills and curriculum’ (e.g. ‘Healthy school policy’, ‘Physical environment’, and ‘School health services’).

#### School physical environment

Twenty instruments included at least 1 subdivision covering ‘School physical environment’, and overall, 32 subdivisions were classified under this component, making it present in more than two-thirds of the instruments. Most subdivisions classified in this component explicitly referred to it in general terms (e.g. ‘Physical environment’ or ‘School physical environment’), with respect to specific structural aspects (e.g. ‘School infrastructure and environmental safety’ or ‘Water sanitation and hygiene’), or in conjunction with ‘School social environment’ (e.g. ‘Environment’ or ‘Safe learning environment’).

Thirteen subdivisions (41%) covering HP activities related to the physical environment explicitly referred to health topics, such as physical activity, nutrition, or social relations and sexuality.

In some occurrences (*n* = 6, 19%), subdivisions intended to cover other components were also classified under ‘School physical environment’ (e.g. ‘Health and well-being policies at school’, ‘Collaboration with the community and services’, or ‘School social environment’).

#### Health services

Eighteen instruments included at least 1 subdivision covering ‘Health services’ and overall 27 subdivisions were classified under this component, making it present in almost two-thirds of the instruments. Half of the subdivisions classified in this component had names explicitly referring to it in general terms (e.g. ‘School health services’ or ‘School healthcare and promotion services’), combined with specific health topics (e.g. ‘Health and nutrition services’ or ‘School counselling, psychological, and social services’), or in conjunction with ‘Community links’ (‘Collaboration with the community and services’).

Seven subdivisions (26%) covering health services explicitly referred to health topics, such as nutrition, mental health, prevention of diseases, smoking, alcohol and drugs, and social relations and sexuality.

In some occurrences (*n* = 4, 15%), subdivisions intended to cover other components were also classified in ‘Health services’ (e.g. ‘School health policy’, ‘School physical environment’, or ‘School social environment’).

#### School social environment

Fourteen instruments included at least 1 subdivision covering ‘School social environment’, and overall, 17 subdivisions were classified under this component, making it the least frequent component. Most subdivisions classified in this component explicitly referred to it in general terms (e.g. ‘School's social environment’ or ‘Social and emotional climate’) or combined with ‘School physical environment’ (e.g. ‘Environment’ or ‘Safe learning environment’) or ‘Individual skills and curriculum’ (‘Appropriate pedagogic methods […] to enhance mental health’).

In some occurrences (*n* = 5, 29%), subdivisions intended to cover other components were also classified under ‘School social environment’ (e.g. ‘Health and well-being policies at school’, ‘Collaboration with the community and services’, or ‘Health skills and competencies’).

### Implementation indicators

Twenty-three instruments included at least 1 subdivision covering dimensions related to implementation indicators, and overall, 79 subdivisions were included in this category (i.e. they were not classified to the HPS components). Fifteen subdivisions (19%) were exclusive to this category.

Examples of activities and processes included steps in the implementation process (e.g. creation of a dedicated team, needs assessment, and training activities) and implementation outcomes (e.g. adherence, dose, participant responsiveness, and quality of delivery). Other aspects were related to the communication and dissemination of the HP activities.

In several occurrences (*n* = 36, 46%), subdivisions intended to cover HPS components were also classified within the implementation indicators (e.g. ‘Healthy school policy’, ‘Community involvement’, and ‘School health services’).

The instrument by Vennegoor *et al*. (2023a) was the only one which explicitly referred to theoretical models and constructs from implementation science by distinguishing various implementation outcomes. Examples of other subdivisions that specifically focused on implementation indicators were found in instruments from Spain (‘The school leads health promotion initiatives through a model of comprehensive health promotion school’; Ministerio de Sanidad and Ministerio de Educación 2023), Portugal (e.g. ‘Teams and levels involved’, ‘Articulation’, or ‘Publicising to the community’; Direção-Geral da Educação 2025), and Italy (‘Vision’, ‘Working process’, and ‘Activities related to HPS Network Membership’; Velasco and Delbosq 2025). The subdivisions classified exclusively in implementation indicators mostly referred to activities related to the steps in the implementation process rather than implementation outcomes.

Activities that targeted school staff as implementers were considered part of implementing other HP activities, while activities aimed at promoting the well-being of the school staff themselves were considered within the components of the approach.

### Health topics

Most instruments consisted of items covering multiple health topics targeting either students, school staff, or both (*M* = 7.1, ranging from 0 to 13). There is a noticeable link between the articulation of an instrument (i.e. number of subdivisions and items) and the number of health topics covered by it (see sheet C of Supplementary File 3).

Supplementary File 5 reports the frequency of instruments with at least one item for each health topic, as well as the HPS components that the items cover for students and school staff. The sheets A and B of the Supplementary File 3 report the results in detail by listing the instruments for students and school staff, respectively. Sheet C of the Supplementary File 3 provides an overview of the number of health topics covered by the instruments. The sheets in the Supplementary File 4 report the classification of health topics for each instrument.

Several topics on student health were covered by the instruments. ‘Nutrition’, ‘Physical activity’, as well as ‘Social relations and bullying’ were present in most instruments. Other health topics, such as ‘Mental health’, ‘Prevention of communicable diseases—hygiene’, and ‘Smoking, alcohol, and drugs’ were also frequently included. In turn, less attention was given to, for instance, ‘Media literacy and use’ and ‘Climate and environmental sustainability’.

Similarly to the subdivision analysis, when considering HP activities within the instruments by health topics, ‘Healthy school policies’ was the most common HPS component, followed by ‘Individual skills and curriculum’.

Several topics on school staff health were covered by the instruments. ‘Social relations and bullying’ was still one of the most present topics, followed by ‘Mental health’, ‘Prevention of communicable diseases—hygiene’, and ‘First aid—medical emergencies’.

School staff was covered less often by HP activities, as demonstrated by a lower frequency for all the health topics considered.

## Discussion

This scoping review aimed to collect and examine instruments measuring the implementation of the HPS approach at the school level. In total, 28 instruments were retrieved. Heterogeneity in instrument length, recipients, data collection methods, theoretical orientations, and methodological quality in the development and validation was observed. In relation to the HPS model, ‘Healthy school policies’ was the most frequent component, while ‘School social environment’ was the least addressed. Implementation indicators (implementation outcomes and steps in the implementation process) were present but were mostly not grounded in theoretical frameworks. Among the health topics, ‘nutrition’ and ‘physical activity’ were most frequently addressed for students, while ‘relationships’ was most often found for school staff.

### Instrument characteristics

One of the research questions addressed in this review regarded the different characteristics of the instruments, such as source, origin, scope, or administration modality. While many were found through the literature review, the expert survey also revealed several instruments not available in databases. This was due to different reasons: some were still under development and could be published later; others have been used within specific HPS networks by operators and institutional stakeholders, and even when publicly available, language barriers limit access for international researchers. This highlights a gap in instrument development and use between practitioners and researchers, related to different purposes. However, many respondents to the expert survey shared their instruments when asked, pointing out the opportunity to strengthen and facilitate cooperative processes among scholars in school HP. Still in relation to these reasons, there were differences in the scope (coverage of HPS components) and methodological quality of the instruments: for instance, only four of them clearly and explicitly reported being evaluated using some form of factorial analysis. Instruments that collected data using more robust, evidence-based indicators and methods (such as following international guidelines, involving institutional entities, or adopting consensus processes during development) were more likely to include all six HPS components considered. This confirms and emphasizes the importance of adopting sound processes to develop and validate instruments measuring the implementation of the HPS approach.

Heterogeneity emerged with respect to administration modality, with both Likert-scale and checklist measures, as well as different recipients, from school members to external operators. The appropriateness or feasibility of these different administration modalities, however, may heavily depend on the context and resources available to researchers.

### Health-Promoting School components: which ones are measured and what needs to be measured

Within the instruments, this review aimed to investigate the measurement of the HPS approach through its components. In different versions of the whole-school approach, a different number of components has been defined and used (WHO 1997, Goldberg *et al*. 2019, WHO and UNESCO 2021b, Lekamge *et al*. 2025a). In this study, a six-component model was adopted, in line with indications from International Union for Health Promotion and Education (IUHPE) and SHE (IUHPE 2008, Vilaça *et al*. 2019). Overall, these HPS components were well-represented in about a third of the instruments found in our study. All components were covered within the instruments, although there were some differences. ‘Healthy school policies’, ‘Community links’, and ‘Individual skills and curriculum’ were the most frequent. ‘Health services’ and ‘School physical environment’ were present in about two-thirds of the instruments, while only half covered ‘School social environment’.

Even though high coverage was reached for ‘Healthy school policies’, only about 15% of those classified subdivisions had names explicitly referring to school policies, and most referred to other HPS components or to health topics. This indicates that, in many cases, the operationalization of HP activities related to other HPS components leads to formulating items investigating policies. This could explain why this component is almost always present within the instruments. Furthermore, the definition of ‘Healthy school policies’ by the SHE Network includes school policies, accepted practices (e.g. routines and habitual procedures), and school planning (Vilaça *et al*. 2019). Having these different HP activities together in one component increased the likelihood of subdivisions to be (also) classified as ‘Healthy school policies’.

While other HPS components were overall well-represented in the instruments, ‘School social environment’ was notably covered by half of the instruments. This may be due to different reasons. Some instruments did not explicitly distinguish between the physical and social environment, giving more attention to the former than the latter (based on the content of the HP activities). While self-reported measures or observations can adequately describe the physical environment (e.g. the presence of facilities and equipment), the social environment is more difficult to measure validly and reliably. It may also depend on the definition of the component (it ‘relates to the quality of the relationships among and between school community members’; Vilaça *et al*. 2019) (Definitions of social environment generally refer to personal relationships and social processes within the neighbourhood or community, although there are differences in what is included [Ontario Agency for Health Protection and Promotion, Public Health Ontario 2024]: therefore, the problem could have occurred regardless of the specific definition by SHE.) used in this analysis. Oftentimes, items related to the social environment as an indirect outcome were operationalized with HP activities falling under other components. In particular, many subdivisions explicitly or implicitly intended to represent the social environment were also (or only) classified in ‘Healthy school policies’ or ‘School physical environment’. Therefore, the definition of the component ‘School social environment’ should be adapted to better stress the actual practices that influence this domain (such as activities that promote inclusion and participation).

Overall, the results showed the importance of considering several components in HPS models. For instance, the three ‘macro-level’ components (School curriculum, Ethos and environment, and Families and communities) conceptualized by Langford *et al*. (2014) were overall covered within the instruments, but the distinction between the six components reported in the results (e.g. the differences in frequency between ‘Community links’ and ‘Health services’) would have been lost. More specific components are useful for defining clearer guidelines to implement the HPS approach and identifying relevant indicators to monitor the implementation.

The results of this review advance the discussion on finding a balance in identifying a set number of HPS components as core indicators, revisiting the model. Indeed, while it is positive that most of the instruments included HP activities related to ‘Healthy school policies’ and ‘Community links’, it should be noted that these components encompass different areas and HP activities. These HP activities may be listed as separate components to better differentiate them. The school’s planning and documents, for instance, could be considered separately. Accepted practices encompass a wide range of HP activities, which can be both the outcomes of policy implementation or routine practices that are not formalized within school documents. Some accepted practices may be better classified when considered within other components, such as the social environment. Further research and theoretical reasoning could explore the possibility of refining what is included in this component.

‘Community links’ was the second most present component. It includes both students’ families and other local stakeholders. These two targets could be perhaps distinguished when considering HPS components, as is done in some specific models (ASCD and CDC 2014). Separating local stakeholders such as vendors, local associations, or local institutions from families could also benefit schools and researchers theoretically and practically (clearly separated scales, standards, and indicators), given that those are two very different groups of stakeholders, similarly to how, within the SHE model, ‘Health services’ is conceptually separated from ‘Community links’, even though they ‘belong’ to the same macro-level (external collaboration).

Another relevant result was that most subdivisions were classified under more than one HPS component or implementation indicator. This is overall theoretically understandable, as HP activities in complex systems imply overlap (Rosas 2017, WHO and UNESCO 2021a): for instance, working on the school playground affects the social environment; implementing a structured curricular programme on drug use prevention may imply training teachers and parental approval. Still, having ‘mixed’ HP activities as indicators of subdivisions related to a specific HPS component might be problematic. The implication is a less valid measure of a specific HPS component.

Conceptual consideration and evidence-based research are needed to further advance this issue of identifying and measuring core components in the HPS approach.

### Health-Promoting School implementation indicators

Together with HPS components, the literature highlights the relevance of investigating implementation indicators (implementation outcomes and steps in the implementation process; Dusenbury *et al*. 2003, Proctor *et al*. 2011, Schaap *et al*. 2018, Vilaça *et al*. 2019). This review also aimed at investigating their presence within instruments. Most of the instruments included subdivisions whose items were related to implementation indicators. The subdivisions classified exclusively in implementation indicators mostly referred to activities related to the steps in the implementation process rather than implementation outcomes. This may be linked to the more attested presence of models of steps in the implementation present within international guidelines (such as Vilaça *et al*. 2019 or WHO and UNESCO 2021b). Despite their presence, implementation outcomes were not included based on a sound theoretical framework. The instrument by Vennegoor *et al*. (2023a) was the only one explicitly rooted in implementation research. Another issue was that these indicators were mostly included in subdivisions which were meant to measure HPS components or health topics. Furthermore, the analysis revealed limited, non-structural attention to the approach’s participatory values, both in terms of guiding activity planning and in involving stakeholders within the school community.

In line with international guidelines recommending the adoption of specific steps for implementing the HPS approach (Vilaça *et al*. 2019, WHO and UNESCO 2021b), future research should more systematically integrate implementation indicators into instruments.

### Health topics: generalizability versus specificity

The final research question of the review concerned the coverage of health topics within instruments. Different health topics were investigated within the instruments, and while highly frequent topics were to be expected (e.g. nutrition, physical activity, social relations and bullying, mental health, and prevention of communicable diseases), others, although important, were less attested (e.g. climate and environmental sustainability and media literacy). Overall, this is not surprising, as the former have historically been considered relevant health topics while the latter gained attention more recently. Furthermore, in some countries, media literacy is a specific school subject, making its inclusion within instruments unnecessary.

While the inclusion of HP activities across different health topics does not directly relate to the quality of the instruments, it could indicate a more detailed investigation of HP activities, collecting evidence with respect to more specific indicators. However, delving into specific topics may take up space that could be used for more general actions that better represent the HPS components. An option to concisely measure (general) HP activities for multiple topics is to provide the topics as a multiple-choice answer, like in the instrument by Vennegoor *et al*. (2023a). On the other hand, having items on specific HP activities for some health topics could be more useful for schools, as they would have clearer guidelines on what to implement.

In some cases, HPS components and health topics were placed at the same level. For instance, the WSCC framework (ASCD and CDC 2014, Lewallen *et al*. 2015), reference for the School Health Index, lists 10 components, but among them it includes: ‘Physical education and physical activity’ and ‘Nutrition environment and services’. These, rather than being wide intervention areas such as the environment or education, are health topics mixed with components. While recognizing the importance of physical activity and nutrition (WHO 1998, 2007, 2022), we argue that mixing health topics and HPS components may lead to confusion and complications with respect to validity and applicability.

### Target groups of health promotion activities

The issue of generalizability and specificity also arose with respect to the target groups of HP activities. In most cases, the HP activities aimed to promote students’ health, while less attention was paid to the school staff. While this study did not have the aim of mapping specific HP activities related to students and the school staff, we differentiated the results when analysing health topics present within the instruments. While some HP activities can be applied both for students and school staff, in most cases, they are specific to students. Furthermore, even if they were applicable to the school staff, not explicitly distinguishing target groups in such HP activities constitutes a limitation within the instruments. Distinguishing specific targets within an equity perspective could also be relevant when investigating HP activities, for instance, with respect to socio-economic status, physical and specific learning disabilities, or migration background (Meroni and Velasco 2023). Further studies could explore this issue.

In some cases, the line between HP activities and ‘instrumental’ activities was blurred. Distinguishing between functional, processual activities that targeted the school staff as a ‘mean’ and HP activities dedicated to the school staff was something that used to be explicitly distinguished in documents and guidelines (e.g. WHO 1998, 2007). Given the approach’s emphasis on HP for all, this distinction is often less explicit.

### Strengths and limitations

Different strengths and limitations of this study should be noted. First, while HPS international guidelines, standards, and indicators exist (Vilaça *et al*. 2019, WHO and UNESCO 2021a), little is known about how they are actually implemented and assessed in real settings. This is, to our knowledge, the first review that summarized the state of the art with respect to quantitative instruments measuring the implementation of the HPS approach at the school level.

Second, relying not only on scientific databases but also on expert knowledge proved to be a successful strategy, as a relevant share of the instruments was retrieved through an expert survey targeting international researchers and practitioners. Using this mixed-method strategy highlighted that oftentimes instruments are not developed for ‘academic purposes’ but are often dedicated towards practice. Therefore, there may be more instruments from other countries and networks that were not included. Including other HP experts and scientific databases could provide more instruments that this study may have missed. Furthermore, modifying the search terms to include other country-specific whole-school approaches may yield additional instruments. Still, this scoping review provides a starting point, collecting both published and unpublished instruments and highlighting the complexity of assessing the implementation of the HPS approach and the need to share information among researchers and practitioners.

A third limitation was not adopting a specific tool to systematically assess the methodological quality of the instruments. However, this was not the aim of the review, and it was not possible to do so in a systematic way, given that many instruments were unpublished or reported little information about their development and use. Further research could gather more information about instrument development methodologies and their use and practical implications within their specific contexts. This could also foster research about the relationship between the implementation of the HPS approach and the role of contextual dimensions. It would also be relevant to systematically investigate how and how much the content validity of the instruments (with respect to components and indicators) relates to their psychometric properties (e.g. factorial validation).

### Implications and insights for future research

This study provides theoretical and practical insights with respect to the development and use of instruments measuring the implementation of the HPS approach.

Based on the retrieved instruments and the data reported in their sources, it was not always feasible to retrieve information about the instruments’ development process or their actual use (e.g. longitudinal evaluation, associations with students’ and staff’s health and social outcomes). Since relatively few instruments included them in a systematic way, future research could focus on developing and validating instruments adopting methodologically sound processes (such as consensus processes and statistical analysis). Furthermore, more research is needed on the actual use of the instruments by researchers and practitioners. Activating collaborative research projects to share theoretical approaches, instruments, data, and contextual information would advance knowledge of the HPS approach implementation.

A relevant challenge in developing instruments is calibrating scope and length. While instruments can be developed for different purposes (e.g. practical purposes, collecting information for administrative records, and research purposes), the scope of the instruments retrieved in this review seemed to indicate that in most cases a comprehensive assessment of HPS implementation was preferred by the developers (e.g. mean of subdivisions within the instruments: 5.8) over the development of short instruments (such as the one by Dadaczynski and Hering 2021). There are clear advantages and disadvantages with respect to long and short instruments. For instance, short instruments are easier to administer, requiring less time and specific knowledge by recipients. They can therefore be more sustainable for schools in long-term monitoring and evaluation studies. On the other hand, longer and more articulated instruments could delve more into the HPS approach, clearly investigating different components with multiple indicators. Furthermore, they could be more tailored to the specifics of the setting (such as country-specific indicators related to health issues and educational systems).

Arguably, collecting more articulated data could provide useful insights into the implementation of the HPS approach. However, country-specific indicators could limit international studies that would further integrate research. Still, by selecting specific HPS components and activities, future research could develop instruments sharing a set of core indicators (Lee *et al*. 2020), reducing the heterogeneity that emerged in this study and allowing international comparisons. Developing short and context-specific ways to measure implementation of the HPS approach is challenging because international instruments should also balance the core components of the HPS approach with the specific characteristics and needs of schools across countries and at different levels. For instance, different health issues (such as environmental and hygiene-related issues) arise in countries with different socio-economic backgrounds. A careful and equity-oriented selection of indicators would be necessary to develop international instruments that foster research. Furthermore, for this very reason, and in order to better identify appropriate and specific indicators in line with the literature, the development of such indicators should take place through activating participatory processes that involve the school community (Gabhainn *et al*. 2023).

## Conclusion

This review streamlines existing efforts to capture the implementation of HPS components for the first time, offering room for consolidation and further development of implementation instruments in this field. A literature review was performed and an expert survey was administered to researchers and practitioners from an international HPS network. Twenty-eight instruments were retrieved from different countries, presenting differences in articulation, size, administration modality, recipients, and methodological quality. Among HPS components, ‘Healthy school policies’ and ‘Community links’ were most frequently covered, while ‘School social environment’ was the least covered. A third of the instruments covered all components.

Items on implementation indicators were overall present but not in a structural and theoretically sound way: instruments should therefore be developed guided by theory on implementation outcomes and processes. The instruments covered several health topics, from well-attested HP topics, such as nutrition and physical activity to new(er) ones, such as environmental sustainability. The results have implications for future researchers to develop appropriate instruments that could both further research and assist practitioners and schools themselves.

## Supplementary Material

daag124_Supplementary_Data

## Data Availability

The data underlying this article will be shared on reasonable request to the corresponding author. With respect to unpublished instruments shared with the authors by health promotion experts, authorization will be asked of them before sharing.
