# Supplementary material for: A scoping review of instruments measuring the implementation of the Health-Promoting School approach
Source: Health Promot Int. 2026 Aug 13;41(4):daag124. doi: 10.1093/heapro/daag124 (PMC13472489; doi:10.1093/heapro/daag124)
Supplement: Supplementary file 1 [file daag124_supplementary_data.zip › Supplementary material file S5. HPS Instruments Review..docx]

Table. Frequency of instruments with at least one HP activity covering health topics for students and school staff and frequency of HPS components by health topic.

| **Health topics** | **Targets** | **Number of instruments** | **HPS components** | | | | | |
| --- | --- | --- | --- | --- | --- | --- | --- | --- |
|  |  |  | **Healthy school policies** | **Community links** | **Health services** | **Individual health skills and action competencies/curriculum** | **School social environment** | **School physical environment** |
| Nutrition | Students | 23 (82%) | 20 (87%) | 6 (26%) | 5 (22%) | 11 (48%) | 3 (13%) | 4 (17%) |
|  | School staff | 6 (21%) | 5 (83%) | 0 (0%) | 1 (17%) | 2 (33%) | 0 (0%) | 0 (0%) |
| Physical activity | Students | 20 (71%) | 18 (90%) | 6 (30%) | 1 (5%) | 13 (65%) | 1 (5%) | 9 (45%) |
|  | School staff | 6 (21%) | 5 (83%) | 0 (0%) | 0 (0%) | 1 (17%) | 0 (0%) | 0 (0%) |
| Smoking, alcohol, and drugs | Students | 17 (61%) | 17 (100%) | 1 (6%) | 4 (24%) | 12 (71%) | 1 (6%) | 3 (18%) |
|  | School staff | 6 (21%) | 5 (83%) | 0 (0%) | 1 (17%) | 2 (33%) | 0 (0%) | 0 (0%) |
| Climate and Environmental Sustainability | Students | 10 (36%) | 9 (90%) | 2 (20%) | 0 (0%) | 4 (40%) | 0 (0%) | 3 (30%) |
|  | School staff | 1 (4%) | 0 (0%) | 0 (0%) | 0 (0%) | 1 (100%) | 0 (0%) | 0 (0%) |
| Prevention of communicable diseases - hygiene | Students | 19 (68%) | 19 (100%) | 3 (16%) | 4 (21%) | 7 (37%) | 2 (11%) | 9 (47%) |
|  | School staff | 9 (32%) | 6 (67%) | 0 (0%) | 0 (0%) | 4 (44%) | 0 (0%) | 2 (22%) |
| Health checkup | Students | 14 (50%) | 10 (71%) | 2 (14%) | 12 (86%) | 1 (7%) | 0 (0%) | 0 (0%) |
|  | School staff | 4 (14%) | 3 (75%) | 0 (0%) | 2 (50%) | 0 (0%) | 0 (0%) | 0 (0%) |
| Sexuality | Students | 14 (50%) | 9 (64%) | 2 (14%) | 4 (29%) | 11 (79%) | 1 (7%) | 3 (21%) |
|  | School staff | 5 (19%) | 2 (40%) | 0 (0%) | 0 (0%) | 3 (60%) | 0 (0%) | 0 (0%) |
| Social relations and bullying | Students | 21 (75%) | 17 (81%) | 6 (29%) | 3 (14%) | 16 (76%) | 13 (62%) | 5 (24%) |
|  | School staff | 16 (57%) | 5 (31%) | 0 (0%) | 0 (0%) | 4 (25%) | 10 (63%) | 0 (0%) |
| Mental health | Students | 19 (68%) | 10 (53%) | 4 (21%) | 11 (58%) | 12 (63%) | 4 (21%) | 2 (11%) |
|  | School staff | 10 (36%) | 5 (50%) | 0 (0%) | 1 (10%) | 5 (50%) | 2 (20%) | 0 (0%) |
| First aid - medical emergencies | Students | 14 (50%) | 10 (71%) | 1 (7%) | 3 (21%) | 7 (50%) | 0 (0%) | 6 (43%) |
|  | School staff | 7 (25%) | 2 (29%) | 0 (0%) | 0 (0%) | 7 (100%) | 0 (0%) | 0 (0%) |
| Traffic safety | Students | 8 (29%) | 7 (88%) | 4 (50%) | 0 (0%) | 3 (38%) | 0 (0%) | 2 (25%) |
|  | School staff | 00 (0%) | 0 (0%) | 0 (0%) | 0 (0%) | 0 (0%) | 0 (0%) | 0 (0%) |
| Media literacy and use | Students | 7 (25%) | 5 (71%) | 0 (0%) | 1 (14%) | 5 (71%) | 1 (14%) | 1 (14%) |
|  | School staff | 3 (11%) | 3 (100%) | 0 (0%) | 0 (0%) | 0 (0%) | 0 (0%) | 0 (0%) |
| General health promotion for school staff | School staff | 12 (43%) | 12 (100%) | 0 (0%) | 0 (0%) | 3 (25%) | 1 (8%) | 2 (17%) |

Percentages in the column “Number of instruments” are computed with respect to the 28 instruments. Percentages in the columns covering the HPS components are based on the column “Number of instruments”.
