# Supplementary material for: A scoping review of instruments measuring the implementation of the Health-Promoting School approach
Source: Health Promot Int. 2026 Aug 13;41(4):daag124. doi: 10.1093/heapro/daag124 (PMC13472489; doi:10.1093/heapro/daag124)
Supplement: Supplementary file 1 [file daag124_supplementary_data.zip › Supplementary material file S6 - HPS Instruments Review.docx]

HPS Instruments Review Supplementary file S6

Note. The information was based on the sources the instruments were retrieved from. It is likely that more information with respect to this information could be available in published sources but its collection is outside the aim of this scoping review.

Table 1. Information about the development and validation of the instruments.

| **#** | **Reporting use of guidelines or other instruments for development** | **Reporting use of pre-testing/piloting** | **Reporting use of reliability measures** | **Reporting use factorial analysis** | **Reporting use of a consensus process (e.g., Delphi)** |
| --- | --- | --- | --- | --- | --- |
| 1 |  |  | ✓ |  |  |
| 2 |  |  | ✓ |  |  |
| 3 | ✓ |  |  |  |  |
| 4 | ✓ |  |  |  |  |
| 5 | ✓ | ✓ | ✓ |  |  |
| 6 | ✓ | ✓ | ✓ |  | ✓ |
| 7 | ✓ | ✓ | ✓ | ✓ | ✓ |
| 8 | ✓ |  |  |  | ✓ |
| 9 | ✓ | ✓ | ✓ |  | ✓ |
| 10 | ✓ |  | ✓ | ✓ | ✓ |
| 11 | ✓ | ✓ |  |  | ✓ |
| 12 | ✓ |  |  |  |  |
| 13 |  |  | ✓ | ✓ |  |
| 14 |  | ✓ | ✓ | ✓ | ✓ |
| 15 |  | ✓ |  |  |  |
| 16 | ✓ | ✓ |  |  | ✓ |
| 17 |  |  |  |  |  |
| 18 |  |  |  |  |  |
| 19 |  |  |  |  | ✓ |
| 20 |  |  |  |  |  |
| 21 |  |  |  |  |  |
| 22 |  |  |  |  |  |
| 23 |  |  |  |  |  |
| 24 | ✓ |  |  |  |  |
| 25 |  |  |  |  | ✓ |
| 26 |  |  |  |  |  |
| 27 |  |  |  |  |  |
| 28 |  |  |  |  |  |
