# Supplementary material for: A scoping review of instruments measuring the implementation of the Health-Promoting School approach
Source: Health Promot Int. 2026 Aug 13;41(4):daag124. doi: 10.1093/heapro/daag124 (PMC13472489; doi:10.1093/heapro/daag124)
Supplement: Supplementary file 1 [file daag124_supplementary_data.zip › Supplementary material file S1 - HPS Instruments Review.docx]

HPS Instruments Review

FIGURES1

Query

(“health promoting school*” OR “health-promoting school” OR “Schools4Health” OR “schools for health” OR “whole-school” OR “whole school” OR “Healthy Primary School of the Future” OR “Comprehensive School Health” OR “healthy school*”)

AND (“implement*” OR “fidelity” OR “indicator*” OR “process” OR “instrument*” OR “evaluat*”)
